# Using Liposomes to Alleviate the Toxicity of Chelerythrine, a Natural PKC Inhibitor, in Treating Non-Small Cell Lung Cancer

**DOI:** 10.3389/fonc.2021.658543

**Published:** 2021-05-27

**Authors:** Jiahui Wang, Yijie Song, Ning Zhang, Ning Li, Congying Liu, Bing Wang

**Affiliations:** ^1^ Experiment Center for Science and Technology, Shanghai University of Traditional Chinese Medicine, Shanghai, China; ^2^ School of Pharmacy, Shanghai University of Traditional Chinese Medicine, Shanghai, China; ^3^ Shenzhen Research Institute, The Hong Kong University of Science and Technology, Shenzhen, China; ^4^ Center for Pharmaceutics Research, Shanghai Institute of Materia Medica Chinese Academy of Sciences, Shanghai, China

**Keywords:** liposome, chelerythrine, non-small cell lung cancer, apoptosis, antitumor effects

## Abstract

**Aim of the Study:**

CHE can inhibit the proliferation of lung cancer cells and induce apoptosis. However, despite having *in vivo* toxicity, CHE has not been thoroughly investigated in term of its *in vivo* antitumor effect. The present study evaluated the antitumor effect of CHE on non-small cell lung cancer cell line HCC827.

**Methods:**

The antitumor effect of CHE on HCC827 was evaluated, and its potential work mechanism was investigated. CHE long circulation liposomes (CHELPs) modified with polyethylene glycol have been optimized and characterized by *in vivo* pharmacokinetic studies. A HCC827 xenograft model was developed on BALB/c nude mice for the assessment of the effects of CHE and CHELP.

**Results:**

CHE might inhibit HCC827 growth through the ROS/PKC-*ϵ*/caspase 3 pathway and glycolysis. The optimized CHELP remained stable after storage for 10 days at 4°C and exhibited sustained drug release, showing approximately one-fifteenth of the *in vivo* clearance rate and 86 times the absorption concentration of free drug. While increasing the bioavailability of CHE, CHELP showed a good therapeutic effect on HCC827 tumor-bearing nude mice and reduced the toxicity of the free drug, improving the safety of CHE.

**Conclusions:**

CHE is a candidate drug for NSCLC, and liposomes are effective in alleviating the toxicity of CHE.

## Introduction

Lung cancer is one of the most common malignancies, with an estimated 1.6 million new cases and 1.38 million deaths each year. Approximately 80–85% of lung cancers are non-small cell lung cancer (NSCLC), and 10−15% are small cell lung cancer (1). Most patients with NSCLC are in the stage of tumor progression, losing early surgery and having a poor clinical outcome (2). Epidermal growth factor receptor-tyrosine kinase inhibitor (EGFR-TKI) is the best treatment for NSCLC with EGFR mutations, and three generations of EGFR-TKIs are currently known, such as gefitinib, afatinib, and osimertinib (3). However, patients often have side effects (4) and drug resistance (5) when treated with EGFR-TKIs, such as EGFR-T790M (6). The effects usually develop in the patients after treatment with first-generation (7) and second-generation (8) EGFR-TKIs. The third-generation EGFR-TKI osimertinib, developed in response to this secondary mutation, has resulted in resistance as well (9). Therefore, discovering medicinal plant materials that are effective in treating NSCLC is of great research value.

Natural herbal medicines with a variety of biological effects have been used in clinical practice for thousands of years. Traditional Chinese medicine, such as astragalus, ginseng, and codonopsis can play an important role in anti-NSCLC treatment through various pathways and targets (10). Chelerythrine (CHE) is one of the main components of *Chelidonium majus* L (11). and can also be extracted from the herbs *Toddalia asiatica* (Linn) Lam (12) and *Macleaya cordata* (Willd.) R. Br (13).. CHEs have shown a variety of effects, including antitumor (14), antibacterial (12), anti-inflammatory (11), pesticidal, and antihepatic fibrosis effects (15) and certain protective effects against diabetic cardiomyopathy (16). In terms of antitumor effect, CHE can inhibit proliferation (17) and induce apoptosis (18) on various human tumor cell lines, such as lung cancer (19), liver cancer (20), breast cancer (14) cell lines, indicating that CHE has great research value and development prospects.

Studies on the CHE treatment of NSCLC cell lines are currently few. He (19) treated NSCLC cells with a combination of CHE and erlotinib, which significantly inhibited proliferation, reduced clone formation, and induced apoptosis. In the research of another NSCLC cell line A549, CHE promoted cell apoptosis and autophagy, decreased cell viability, and induced cell death by increasing ROS generation (21). However, the *in vivo* antitumor effect of CHE has not been comprehensively researched, and CHE has been found to have a certain level of toxicity to animals (22).

Therefore, in this paper, the effects of CHE on cancer cell line and normal cell lines were respectively evaluated, and the potential pathways were investigated. For the reduction of CHE toxicity, the liposomes of CHE (CHELP) were prepared for the treatment of NSCLC cell line HCC827 with EGFR mutation. Moreover, polyethylene glycol (PEG) was introduced to long circulation liposomes to enhance the hydrophilicity of liposomes and promote a sustained release effect (23). The study may offer reference for formulating novel drug delivery systems using traditional Chinese medicine for cancer treatment.

## Materials and Methods

### Materials

Chelerythrine was purchased from Chengdu Must Biotechnology Co., Ltd (Chengdu, China). Chelerythrine reference substance and propranolol reference substance were purchased from the National Institute for the Control of Pharmaceutical and Biological Products (NICPBP, China). 1,2-distearoyl-sn-glycero-3-phosphatiylethanol-amine-N-[methoxy(poly-ethyleneglycol)2000] (DSPE-PEG2000), cholesterol (CHOL) and hydrogenated soybean phosphatidylcholine (HSPC) were purchased from A.V.T. pharmaceutical Co., Ltd (Shanghai, China). Dimethyl sulfoxide (DMSO), DCFH-DA, and 3-(4,5-dimethylthiazol-2-yl)-2,5-diphenyltetrazolium bromide (MTT) were purchased from Sigma Aldrich (USA). PI, RNase, Mitochondrial Membrane Potential Assay Kit with JC-1, Caspase 3 viability kit, and SDS-PAGE gel rapid preparation kit were purchased from Beyotime Biotechnology Co., Ltd (Shanghai, China). FITC Annexin V Apoptosis Detection Kit I was purchased from BD Biosciences (USA). Trans-well chamber was purchased from Corning Co., Ltd (Shanghai, China). Cell counting kit-8 (CCK-8), methanol and crystal violet staining solution were purchased form BBI Life Sciences Co., Ltd. XF Base Medium, XF24 Cell Culture Microplates, XF24 Extracellular Flux Assay Kits, XF Calibrant, XF Cell Mito Stress Test Kit were all purchased from Agilent technologies Inc. (Shanghai, China). L-Glutamine, Sodium pyruvate solution, D-glucose were purchased from Sigma (USA).18 ICR mice and 26 BALB/C nude mice were purchased from Jiangsu GemPharmatech Co, Ltd and raised in Laboratory Animal Center, Shanghai University of Traditional Chinese Medicine.

### Cell Culture

The human non-small cell lung cancer cell line HCC827 was obtained from Chinese Academy of Sciences Cell Bank (Shanghai, China). Cells were cultured in RPMI medium 1640 (Hyclone, USA) with 10% FBS (Lonsera, USA) at 37°C in a humidified atmosphere with 5% CO_2_.

### Cell Viability Assay

Cell counting kit-8 (CCK-8) was applied to assess cell proliferation following the manufacturer’s instructions (BBI Life Sciences, E606335-0500). In brief, HCC827 cells were seeded into a 96-well plate at a density of 8,000 cells/well in 100 μl cell culture medium with different contents of CHE (0, 5, 10, 15, 20, 30, and 40 μM) respectively. Then, cell proliferation was detected by microplate reader at 450 nm wavelength (Biotek, EPOCH2) on days 1, 2, and 3 after adding 10 μl CCK8 reagent in each well and incubating cells in darkness for 1 h. The rate of cell proliferation was calculated by the optical density. Moreover, another two cell lines (HL-7702 and HEK-293) were also assessed by the same way in order to understand the impact of CHE on normal cells.

*In vitro* cytotoxicity of chelerythrine was evaluated in HCC827 cell line using MTT assay. Cells were seeded in 96-well plates at a density of 8,000 cells/well and treated with 100 μl of chelerythrine in full medium of different concentrations for 24 h. Then 20 μl MTT (5 mg/ml) was added and incubated for 4 h at 37°C. The formed formazan salt was dissolved in 100 μl of DMSO and absorbance was detected at 490 nm using a plate reader (SpectraMax M5, Molecular Devices). Data were represented as means ± standard deviation (SD). The mean drug concentration required for 50% growth inhibition (IC50) was determined using Graphpad Prism 7.0 software.

### Apoptosis and Cell Cycle Analysis

Briefly, HCC827 cells were seeded in six-well plates 24 h prior to drug treatment. In the apoptosis assay, apoptotic populations of vehicle- or chelerythrine-treated cells were quantified using the dual staining FITC-Annexin V Apoptosis Detection Kit as per manufacturer’s instructions. After the drug incubation, the supernatant was collected in the tube, and cells were collected using 0.25% Tyrisin without EDTA in flow tube as well. Cells were washed by PBS twice before being stained with FITC dyeing solution in darkness for 30 min and then stained with PI dyeing solution for 10 min, waiting for flow cytometry on ice. In cell cycle assay, after incubation with RNase A (5 μg/ml) for 30 min, cells were only stained with PI solution (20 μg/ml) for 10 min and also detected by FACS Calibur flow cytometer (BD Biosciences).

### Wound-Healing and Trans-Well Migration Assays

In wound-healing assay, cells were seeded into six-well culture plates after counting. When the cells grew to an appropriate density, a straight scratch was made by a pipette tip in the cell layer. Subsequently, the floating cells were removed, and new medium containing a different concentration of CHE was added to the culture for 24 h. The distance between the wounded area was measured by using the inverted fluorescence microscope (OLYMPUS, CKX41) at 0, 12, and 24 h to assess the wound-healing.

Trans-well assay was used to evaluate the migration of cells. In short, 100 μl counted cell suspension was added into each trans-well chamber (Corning). 600 μl medium containing 20% FCS was added in the wells of 24-well culture plate as a bottom culture medium to induce cell migration. The cells were incubated for 24 h before the trans-well chambers were taken out, and the medium was discarded. Trans-well chambers were washed twice with PBS. The cells were fixed *via* formaldehyde for 30 min, then air-dried, and stained with 0.1% crystal violet for 30 min, and non-migrated cells were gently wiped off the top-well with a cotton swab. After washing the trans-well chambers three times with PBS, the number of migrated cells was counted in three randomly-selected fields under a 400-fold microscope per well to assess the cell invasion capability.

### Extracellular Acidification Rate Test

Cancer cells under a hypoxic environment mainly depend on glycolytic pathways as a source of cell energy. Therefore, extracellular acidification rate (ECAR) was measured by a Seahorse XF24 Extracellular Flux Assay Kits (Agilent technologies, Q29216) and Seahorse XF24 Extracellular Flux Analyzers (Agilent technologies) in order to detect glycolysis. HCC827 cells were planted in a Seahorse XF24 Cell Culture Microplates (Agilent Technologies, 28816) at a density of 1 × 10^5^ cells/well. 1 mL hydration fluid was added into the lower layer of the XF24 Extracellular Flux Assay Kit and hydrated overnight in a CO_2_-free incubator at 37°C. Meanwhile, the drug and Seahorse XF basic medium (Agilent technologies, 102353) were prepared, of which the pH value was adjusted to 7.4. The medium was put into 37°C water bath for 1 h before washing the cells twice with it. Then each well was replenished by 450 μl Seahorse XF basic medium. Culture cells for another 1 h at 37°C. Different concentrates of CHE were diluted, and 75 μl solution was added into the upper layer of XF24 Extracellular Flux Assay Kits for measurements. After 30 min, the lower layer of XF24 Extracellular Flux Assay Kits was removed and replaced by the cell plate for detecting.

### ROS Generation and Mitochondrial Membrane Potential Analysis

Briefly, the cells were seeded in 12-well plates 24 h prior to drug treatment. In ROS generation analysis, cells were stained with 10 μM DCFH-DA solution for 30 min in the dark after CHE treatment for 2 h and then collected. After being washed by PBS twice, dihydrodichlorofluorescein (DCF) fluorescence was analyzed by FACS Calibur flow cytometer (BD Biosciences). In mitochondrial membrane potential (*Δψ*m) analysis, cells were collected and stained with JC-1 dyeing solution after CHE treatment for 2 h. Then cells were detected by flow cytometer as well. The change of MMP could be obtained by calculating the ratio of fluorescence degree between the FL2 channel and FL1 channel.

### PKC-ϵ and Caspase 3 Expression Analysis

Briefly, cells were seeded in six-well plates 24 h prior to CHE drug treatment. PMA, the PKC activator was added with the high dose group to verify the mechanism. After 4 h, cells were collected for PKC-*ϵ* and caspase 3 expression analysis through western blot assay and caspase 3 viability kit, respectively. Precooled RIPA lysate with 1% PMSF was added and centrifuged to get the supernatant for the following western blot assays to evaluate the expression of PKC-*ϵ* in cells. For caspase 3 viability detection, after being ice bath cracked for 30 min, caspase 3 viability in the samples could be directly detected at 405 nm using a plate reader (SpectraMax M5, Molecular Devices).

### Optimization and Characterization of CHELP

CHELP was prepared by the thin-film hydration followed by hydration method. Briefly, a certain amount of CHOL, DSPE-PEG2000, HSPC (1:1:3, w/w/w) was dissolved completely in ethanol and evaporated to get a thin film. Then (NH4)_2_SO_4_ buffer solution was added for hydration, lasting 45 min, and blank liposomes were obtained after ultrasonication by a probe sonicator (Qsonica sonicators, America). CHE water solution was added into blank liposomes, and NaHCO_3_ buffer solution was used to adjust the pH value to 8.0, stirring for 30 min to get the CHE liposomes. Finally, unbound CHE was removed by gel permeation chromatography on Sephadex G-50.

CHELP was optimized using the central composite design/response surface method. The factors, CHOL : HSPC (w/w) (A), CHE–lipid ratio (B), and the drug incubation time (C) were investigated. The particle size (Y1) and drug loading (DL) (Y2) were used as the indexes. The three levels of low, medium and high were set for the investigation factors (encoding −1, 0, and 1 respectively; **Table 1**). The design-expert 8.0.6 software was used for the experimental design, and 20 sets of experimental plans were generated.

**Table 1:** Factors and levels.

Factors	Levels		
	−1	0	1
A	0.33	1.67	3
B	0.06	0.16	0.25
C	15	30	45

### *In Vitro* Release of CHE From CHELP

*In vitro* release analysis of CHE liposomes was performed by the dialysis method. Briefly, 1 ml CHE liposomes injected into a dialysis bag (14,000 Da, molecular weight cut-off) was sunk into 35 ml PBS (pH 5.6) and PBS (pH 7.4) at 37°C, and stirred at the speed of 150 rpm. At regular intervals, 1 ml CHE containing simulated PBS solution was taken out, and 1 ml fresh PBS solution was added. The aliquots were filtrated with 0.45 μm Millipore filtration before injected into the HPLC system.

### *In Vivo* Pharmacokinetic Study of CHELP

*In vivo* pharmacokinetic study of CHELP was conducted in ICR mice (20–25 g). Eighteen mice were randomly divided into two groups and received i.v. administration of CHE and CHE liposomes at a dose of 6 mg/kg. After administration, blood was collected through veins at 2, 5, 15, and 30 min, 1, 2, 4, 8, and 24 h into centrifuge tubes with heparin sodium. The blood samples were subsequently centrifuged at 3,000 rpm for 10 min to obtain the plasma.

To detect the CHE content in the plasma, 400 μl internal standard (I.S.) propranolol-containing methanol-acetonitrile (1:1) was added into 50 μl plasma. In order to precipitate protein, the mixture was vortexed and centrifuged at 13,000 rpm for 15 min. 250 μl supernatant was taken for nitrogen blowing and redissolved with 100 μl 70% acetonitrile. After centrifugation at 13,000 rpm for 15 min, the supernatant was detected by Angilent 6470 Triple Quad LC/MS.

The Angilent 1290 Infinity II UPLC system was applied with an ACQUITY UPLC BEH C18 Column (2.1 × 100 mm, 1.7 μM). Mobile phase: A: 0.1% Formic Acid; B: Acetonitrile. The LC gradient elution was programmed as **Table 2**. Column temperature: 30℃; Flow rate: 0.3 ml/min; Injection volume: 1 μl. The MS condition for CHE and I.S. quantification of Angilent 6470 Triple Quad LC/MS with electrospray ionization is shown in **Table 3**. Ion source: electrospray ion source (ESI); Detection method: positive ion; Monitoring mode: MRM; Test time: 5 min; Gas temp: 300°C; Gas flow: 5 L/min; Nebulizer: 45 psi; Capillary voltage: 3,500 V.

**Table 2:** The LC gradient elution.

Time	A %	B%
0.00	75.00	25.00
1.00	75.00	25.00
1.50	55.00	45.00
3.50	55.00	45.00
4.00	75.00	25.00
5.00	75.00	25.00

**Table 3:** Parameters of ESI-MS/MS.

Compound name	Precursor ion	Product ion	Dwell	Fragmentor	Collision energy	Polarity
Chelerythrine	348.4	318.0	150	50	20	Positive
Propranolol	260.2	116.3	150	110	14	Positive

### Animals and HCC827 Xenograft Model

Male BALB/c nude mice (18–20 g) were kept in an experimental animal barrier environment for one-week adaptive feeding. 26 mice were subcutaneously injected at the right flank with 0.2 ml HCC827 cells (1:1, with Matrigel). Tumor volume was calculated as the formula below.

$$\text{Tumor}\;\text{volume}\;(\text{m}{\text{m}}^{3})=\frac{1}{2}\times \text{length}\times \text{widt}{\text{h}}^{2}$$

### *In Vivo* Antitumor Effect Evaluation

When the tumor volume arrived 80–150 mm^3^, mice were randomly divided into three groups: control group (normal saline, n = 8), CHE group (10 mg/kg, n = 10), CHELP group (10 mg/kg, n = 8). The mice were treated through the tail vein once a week, four times in total, lasting for 23 days. The control group was injected with the same volume of normal saline. The tumor volume and the body weight of mice were recorded twice a week.

### Tunel Assays and ROS Detection of Tumor Tissues

After 23 days, mice were sacrificed, and tumors were collected. Parts of the tumors were fixed in 4% paraformaldehyde. The following routine pathological sections were performed, and the tissues were cut, dehydrated, transparent, wax-permeable, and embedded. Then TUNEL staining was conducted to evaluate the apoptosis effect of CHE and CHELP, and parts of the tumor tissues were stored in dry ice for the ROS detection using superoxide anion fluorescent probe and DAPI.

### PKC-ϵ Expression Analysis in Tumor Tissues

After 23 days, mice were sacrificed, and tumors were collected. Parts of the tumors were homogenized with RIPA lysis buffer with 1% PMSF and centrifuged to get the supernatant for the following western blot assays to evaluate the expression of PKC-*ϵ* in tumors.

### Safety Evaluation and Histopathological Analysis

After 23 days, mice were sacrificed and heart, liver, spleen, lung, and kidney were collected. The spleen morphology and its organ coefficient were recorded. Heart, liver, spleen, lung, and kidney were fixed in 4% paraformaldehyde. The following routine pathological sections were performed, and the tissues were cut, dehydrated, transparent, wax-permeable, and embedded. Then the HE staining was conducted for safety evaluation.

### Statistical Analysis

The experimental data were represented as means ± standard deviation (SD), and they were processed with GraphPad Prism 7.0 software, using one-way ANOVA or two-way ANOVA analysis.

## Result

### CHE Suppressed the Cell Proliferation of HCC827 but Not That of HL-7702 and HEK-293

**Figure 1:**
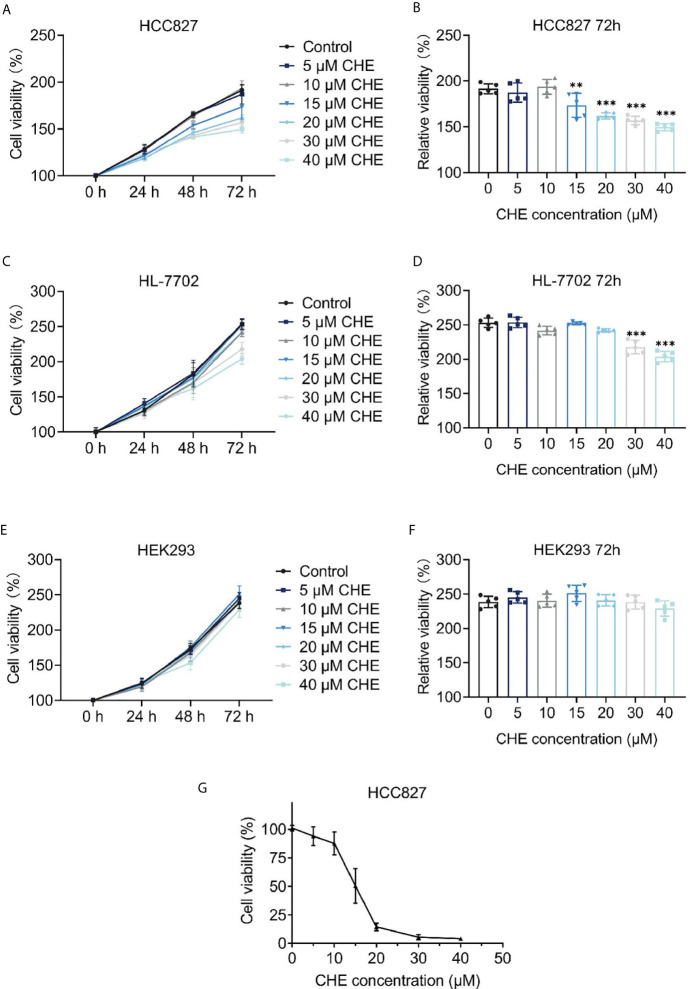
The cell proliferation of HCC827, HL-7702, and HEK-293 after treatment with CHE. **(A, C, E)** Cell viability of HCC827, HEK-293, HL-7702 cells after treatment with CHE through the CCK-8 assay, respectively. CHE induced the apoptosis of HCC827 cells. **(B, D, F)** The relative viability of HCC827, HEK-293, HL-7702 cells after treatment with CHE at 72 h, respectively. **(G)** The MTT result of HCC827 after treatment with CHE. (***p < 0.001, **p < 0.01).

The cytotoxicity of CHE was evaluated through the CCK-8 assay, which was carried out on HCC827, HL-7702, and HEK293 cells. As shown in **Figures 1A, B**, CHE successfully inhibited the proliferation of HCC827 cells *in vitro* in a dose-dependent manner when the content of CHE was greater than 15 μM, and the inhibitory effect was amplified over time. However, CHE rarely affected the cell viability of HEK-293 cells, and there was no significant difference between any group and group with no CHE (**Figures 1E, F**). Only high-dose CHE groups (30 and 40 μM) showed significant differences compared with the control group in terms of the proliferation of human liver cells (HL-7702) at 72 h (**Figures 1C, D**), indicating that CHE has a specific toxicity to cancer cells, and a high dose might lead tissue-specific normal cell damage. Moreover, the antitumor effect of CHE on HCC827 was confirmed by the MTT result (**Figure 1G**), and the IC50 value of CHE was 15.13 ± 0.93 µM for HCC827.

### CHE Inhibited Growth of HCC827 Cells and Induced Apoptosis

**Figure 2:**
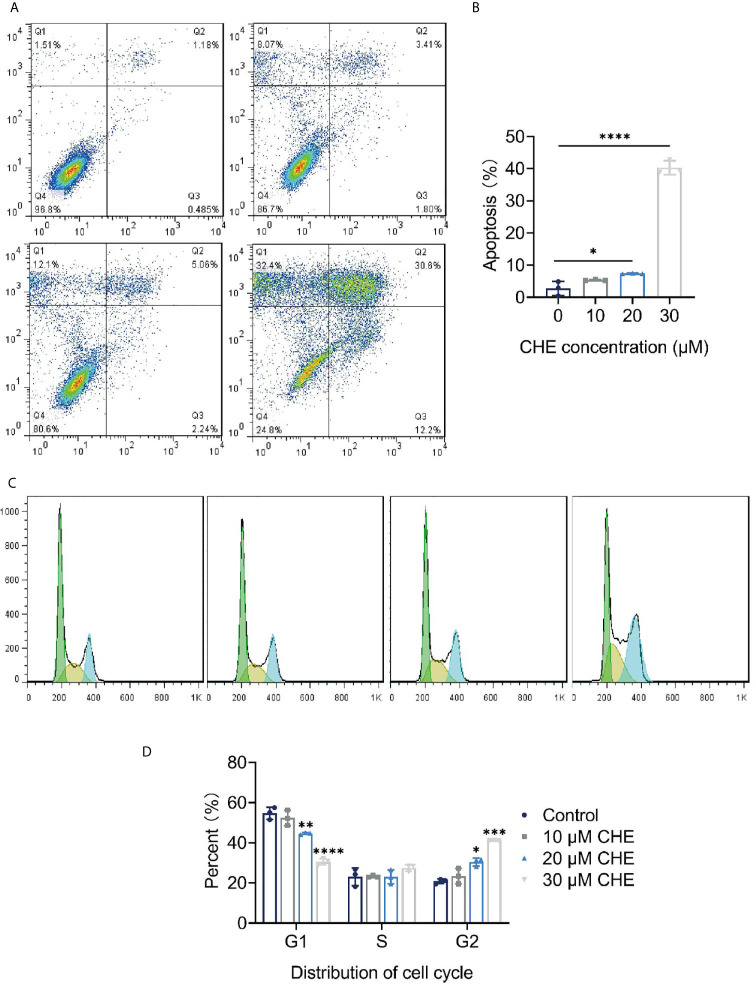
CHE inhibited growth of HCC827 cells and induced apoptosis. **(A, B)** CHE induced the apoptosis of HCC827 cells. **(C, D)** CHE arrested HCC827 in G2/M phase. (****p < 0.0001, ***p < 0.001, **p < 0.01, *p < 0.05).

The apoptosis and cell cycle of HCC827 cells were analyzed after CHE treatment. CHE showed a good effect in inducing apoptosis in HCC827 cells (**Figures 2A, B**). When the cells were treated with 30 µM CHE, the apoptosis rate increased significantly, reaching approximately 40%. Notably, the cell cycle was arrested in the G2/M phase (**Figures 2C, D**). With increasing CHE dose, the proportion of HCC827 cells in the G1 phase decreased, and the proportion in the S phase nearly did not change. By contrast, the proportion in the G2 phase increased significantly, and this increase inhibited the growth of cancer cells (24).

### CHE Impeded the Migration and Invasion of HCC827 Cells

**Figure 3:**
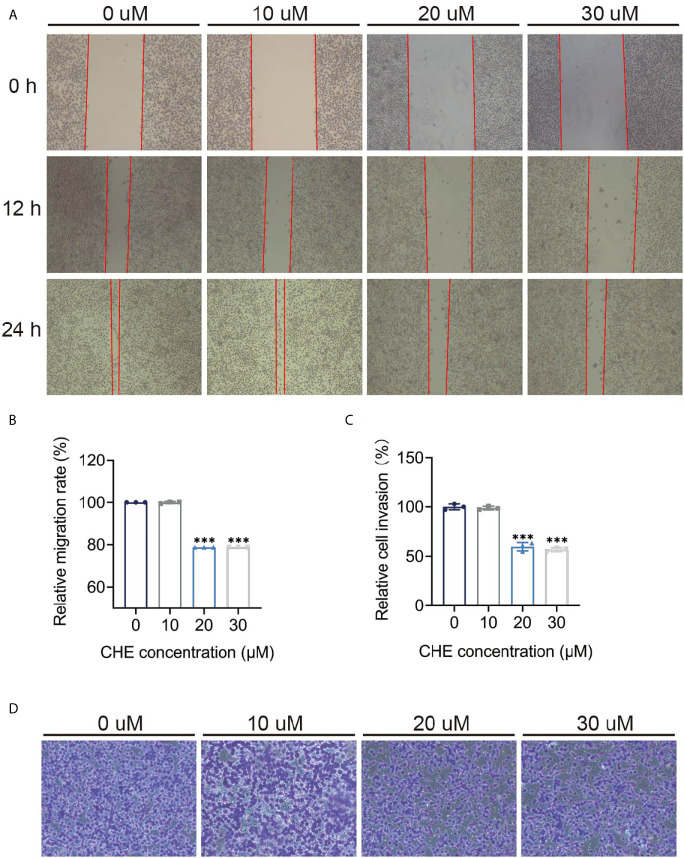
CHE impeded the migration and invasion of HCC827 cells. **(A, B)** The wound-healing assays of HCC927 cells after treatment with CHE. **(C, D)** The trans-well migration assays. (***p < 0.001).

The inhibitory effect of CHE on the activity of HCC827 cells was evaluated through wound-healing and trans-well migration assays. The results of the wound-healing assay revealed that the distance between the scratch borders significantly increased after the HCC827 cells were treated with 20 or 30 μM CHE for 12 and 24 h compared with the control group or low-dose CHE group (10 μM), and the scratch borders after 24 h were nearly indistinguishable (**Figures 3A, B**). Consistent with the outcomes of the wound-healing assay, the invasion activity of HCC827 was suppressed in the presence of 20 or 30 μM CHE as confirmed by the trans-well migration assay (**Figures 3C, D**). The number of migratory cells significantly decreased after incubation with 20 or 30 μM CHE compared with the number in the control but showed no obvious difference from the number in the group with 10 μM CHE. Overall, our results suggested that CHE depresses the migration and invasion of HCC827 cells.

### CHE Reduced the Glycolysis Capacity of HCC827 Cells

**Figure 4:**
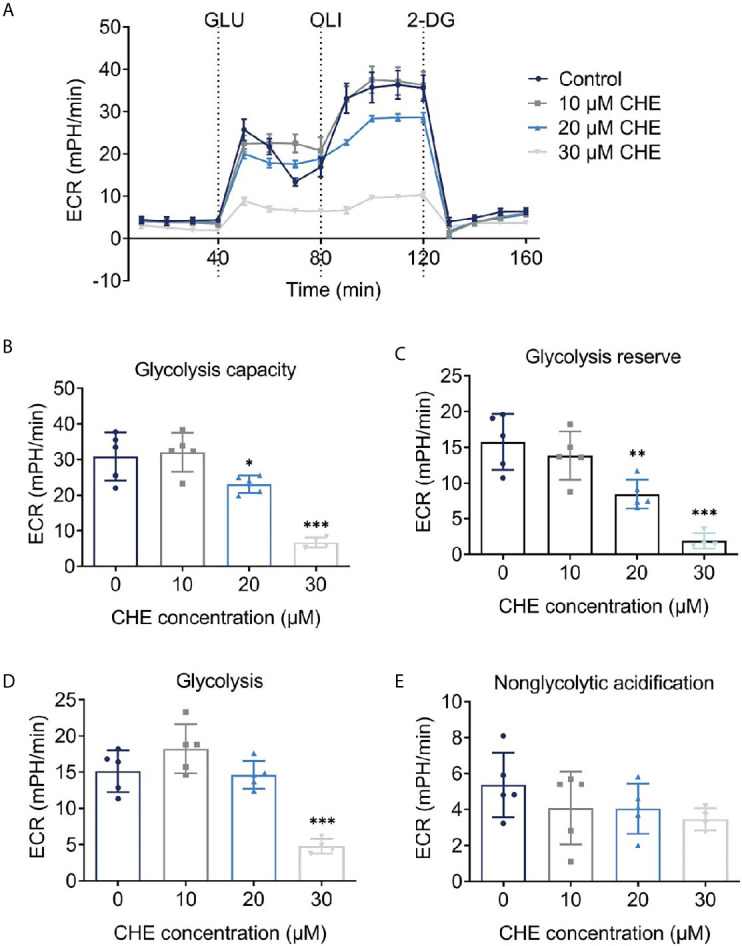
CHE reduced the glycolysis capacity of HCC827 cells. **(A)** Extracellular acidification rate (ECAR) test of HCC827 cells after treatment with CHE; **(B)** Glycolysis capacity; **(C)** Glycolysis reserve; **(D)** Glycolysis; **(E)** Non-glycolytic acidification. (***p < 0.001, **p < 0.01, *p < 0.05).

As the main energy pathway of tumor cells, glycolysis level may reflect inhibited growth. Logically, the overall glycolysis level of HCC827 changed after treatment with CHE, and glycolysis was significantly inhibited by 30 μM CHE (**Figure 4A**). Compared with the control group, CHE-treated group showed decreased glycolysis, glycolysis capacity, glycolysis reserve, and non-glycolytic acidification (**Figures 4B–E**), and 30 μM CHE exhibited the most significant inhibitory effect on HCC827 cells (***p < 0.001). This result showed that the metabolism and mitochondrial respiration of HCC827 might be down-regulated by CHE and limit the activity of HCC827.

### CHE Affected *Δψ*m and ROS on HCC827 Cells

**Figure 5:**
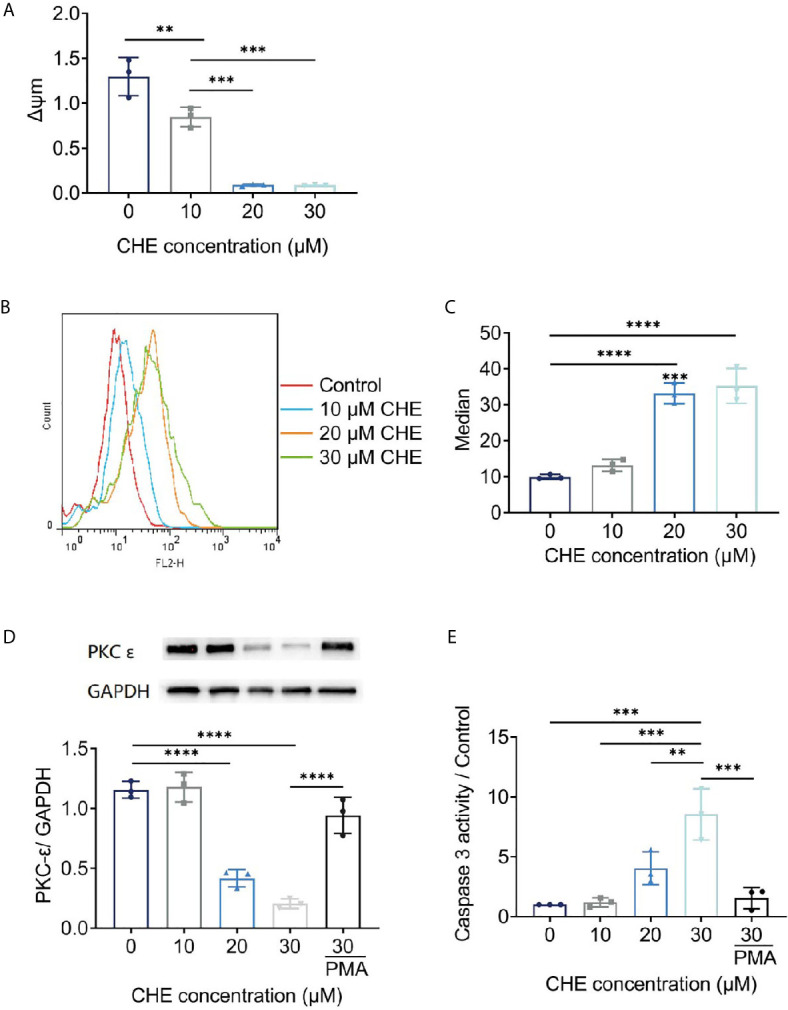
The potential mechanism of HCC827 cell’s apoptosis induced by CHE. **(A)** The *Δψ*m of HCC827 decreased after treatment of CHE. **(B, C)** ROS level in HCC827 cells after treatment of CHE. **(D)** CHE inhibited the expression of PKC-*ϵ* in HCC827 cells. **(E)** CHE increased of viability of caspase 3 in HCC827 cells. (****p < 0.0001, ***p < 0.001, **p < 0.01).

Antitumor effects were linked to decrease in *Δψ*m and increase in ROS generation. The fluorescence value of FL2 channel decreased continuously, and the ratio of the fluorescence degree between the FL2 and FL1 channels decreased with increasing dose. The *Δψ*m of HCC827 cells were decreased by CHE significantly, leading to cell apoptosis (**Figure 5A**) (25). After treatment with CHE, intracellular ROS increased (**Figures 5B, C**), which can produce cytotoxicity by acting on proteins and damaging DNA and mediate the rapid apoptosis of cells (26, 27).

### CHE Influenced the Expression of PKC-*ϵ* and Caspase 3

CHE might act on the ROS/PKC-*ϵ*/caspase 3 pathway, inducing cell apoptosis and inhibiting the growth of tumor cells. CHE, as a kind of PKC inhibitor, showed great antitumor effects. Caspase 3 is one of the important indicators of the early apoptosis of cells and can reflect the pro-apoptotic effect of drugs on cells and participate in the regulation apoptosis through various pathways (28, 29). Our results showed that CHE can induce early apoptosis, significantly inhibit PKC-*ϵ* (**Figure 5D**), and promote the expression of caspase 3 in a dose-dependent manner (**Figure 5E**). When the high-dose group (CHE 30 µM) was co-treated with PKC activator PMA, the expression of PKC-*ϵ* increased significantly, and the expression of caspase 3 decreased significantly as shown in **Figures 5D, E**. Moreover, CHE can induce early apoptosis by inhibiting protein PKC-*ϵ*. After PMA was added, the viability of caspase 3 decreased, and thus the apoptosis-inducing effect of CHE on non-small cell lung cancer cell HCC827 was blocked.

### Optimization of CHELP

As shown in **Table 4**, the influence of three factors (A: CHOL : HSPC ratio; B: CHE drug–lipid ratio, and C: drug incubation time) and five levels on the response variables (Y1: particle size and Y2: drug loading) were studied. The quadratic polynomial regression equation was fitted using Design Export 7.0 software (Stat-Ease Inc., Minneapolis, MN, USA).

**Table 4:** Design and results.

Run	F1 CHOL: HSPC ratio	F2 CHE drug-lipids ratio	F3 drug incubation time	Response 1 Size (nm)	Response 2 DL (%)
1	1.67	0.16	30.00	127.9	12.927
2	1.67	0.16	30.00	119	13.1154
3	1.67	0.16	30.00	137.5	11.2043
4	2.46	0.21	38.92	154	10.7668
5	3.00	0.16	30.00	240.6	9.32359
6	0.87	0.10	21.08	118.8	9.31382
7	1.67	0.16	30.00	123.1	13.7509
8	2.46	0.21	21.08	204	15.6274
9	1.67	0.16	30.00	117.8	11.8688
10	0.33	0.16	30.00	109.9	15.9702
11	1.67	0.16	45.00	143.8	7.15909
12	1.67	0.16	30.00	146.3	14.3151
13	0.87	0.21	21.08	126.7	15.7016
14	2.46	0.10	21.08	196.9	5.96926
15	1.67	0.06	30.00	125.2	3.93899
16	1.67	0.25	30.00	158.1	18.3233
17	0.87	0.10	38.92	123.4	6.77879
18	0.87	0.21	38.92	138	13.4262
19	2.46	0.10	38.92	171.7	2.79281
20	1.67	0.16	15.00	144.8	10.6013

According to the variance analysis of particle size (**Table 5**), particle size model F was 14.06 (p < 0.0001), indicating that the regression equation fitted well. The lack of fit, p = 0.3314 (>0.05), was non-significant, indicating the absence of abnormal points in the data. According to the results of particle size variance analysis, CHOL : HSPC (w/w) (A), drug fat ratio (B), and incubation time (C) were fitted by multiple linear regression and quadratic polynomial equation. Regression equation: Y1 = 128.63 + 32.18·A + 4.92·B − 4.47·C − 4.14·AB − 11.39·AC − 2.26·BC + 16.27·A^2^ + 4.39·B^2^ + 5.32·C^2^ (p = 0.0001, significant; Lack of Fit, p = 0.3314, not significant; R^2^ = 0.9268).

**Table 5:** Results of variance analysis of size (Y1).

Source	Sum of squares	df	Mean square	F-value	p-value	
Model	20,064.19	9	2,229.35	14.06	0.0001	Significant
A—CHOL : HSPC ratio	14,144.50	1	14,144.50	89.20	<0.0001	
B—drug–lipid ratio	330.97	1	330.97	2.09	0.1791	
C—drug incubation time	272.30	1	272.30	1.72	0.2193	
AB	136.95	1	136.95	0.86	0.3746	
AC	1037.40	1	1037.40	6.54	0.0285	
BC	40.95	1	40.95	0.26	0.6223	
A^2	3813.63	1	3813.63	24.05	0.0006	
B^2	277.48	1	277.48	1.75	0.2153	
C^2	408.63	1	408.63	2.58	0.1395	
Residual	1,585.69	10	158.57			
Lack of fit	953.65	5	190.73	1.51	0.3314	Not significant
Pure error	632.04	5	126.41			
Cor total	21,649.88	19				

According to the variance analysis of drug loading (**Table 6**), the drug loading model F was 29.20 (p < 0.0001), indicating that the regression equation fitted well. The lack of fit, p = 0.5754 (>0.05), was non-significant, indicating the absence of abnormal points in the data. CHOL : HSPC (w/w) (A), drug fat ratio (B), and drug incubation time (C) were fitted by multiple linear regression and quadratic polynomial equation. Regression equation: Y2 = 12.89 − 1.56·A + 4.02·B − 1.36·C + 0.57·AB − 0.40·AC − 0.18·BC − 0.23·A^2^ − 0.76·B^2^ − 1.56·C^2^ (p < 0.0001, significant; Lack of Fit, p = 0.5754, not significant; R^2^ = 0.9633).

**Table 6:** Results of variance analysis of drug loading efficiency (Y2).

Source	Sum of squares	df	Mean square	F-value	p-value	
Model	323.52	9	35.95	29.20	<0.0001	Significant
A—CHOL : HSPC ratio	33.04	1	33.04	26.84	0.0004	
B—drug–lipid ratio	220.36	1	220.36	179.00	<0.0001	
C—drug incubation time	25.43	1	25.43	20.66	0.0011	
AB	2.64	1	2.64	2.15	0.1737	
AC	1.30	1	1.30	1.06	0.3281	
BC	0.25	1	0.25	0.21	0.6596	
A^2	0.75	1	0.75	0.61	0.4527	
B^2	8.42	1	8.42	6.84	0.0258	
C^2	35.07	1	35.07	28.49	0.0003	
Residual	12.31	10	1.23			
Lack of fit	5.61	5	1.12	0.84	0.5754	Not significant
Pure error	6.70	5	1.34			
Cor total	335.83	19				

R^2^ in the regression equation represents a linear relationship. The closer the value is to 1, the closer the predicted value of the regression model is to the real value, that is, the stronger the predictive power is. In the above two equations, R^2^ is greater than 0.9, and the mean p value of the unfitting term is greater than 0.05, indicating that the model fits well.

**Figure 6:**
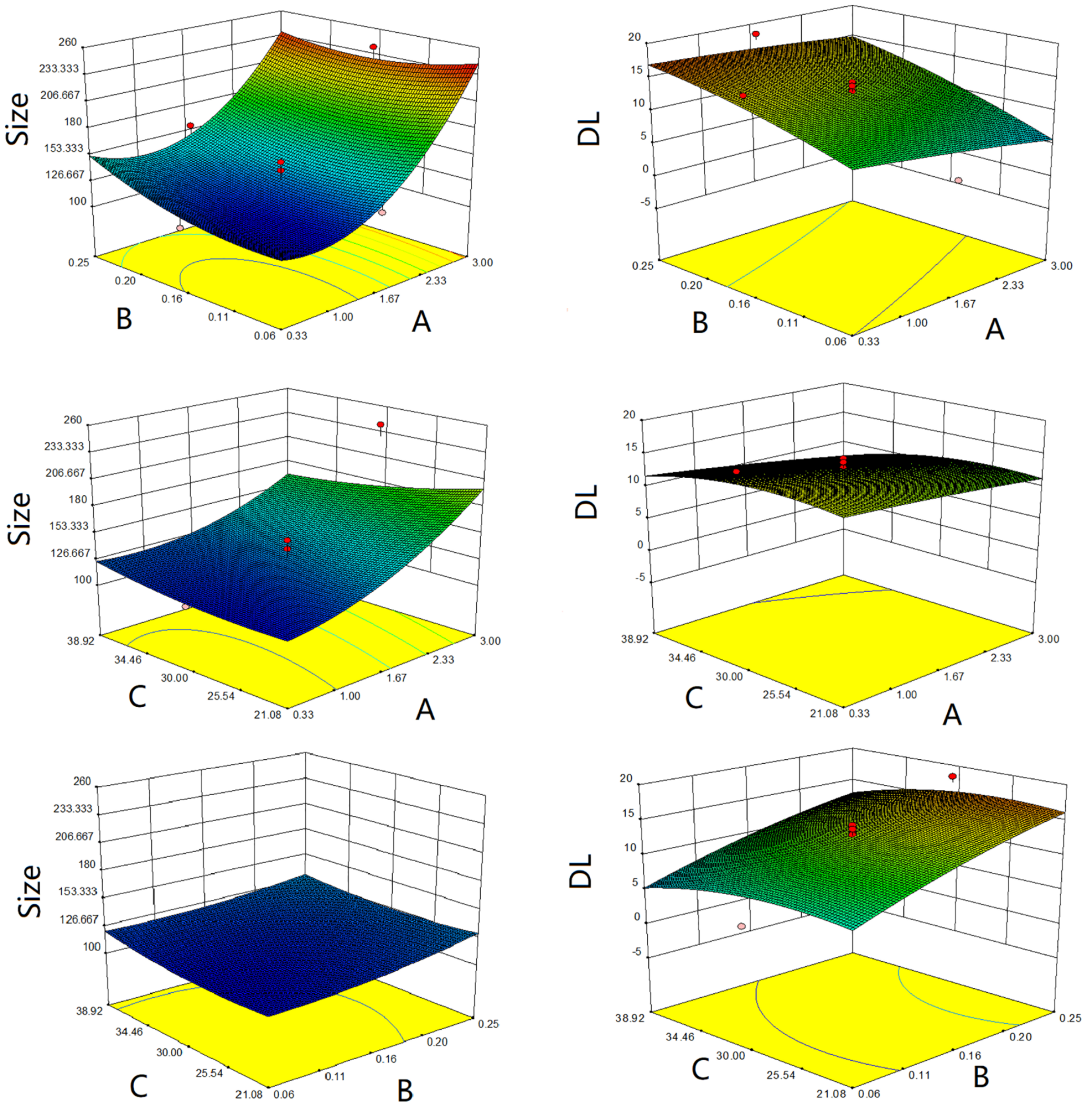
Response surface diagrams of CHOL : HSPC ratio **(A)**, CHE drug–lipid ratio **(B)** and drug incubation time **(C)** to size and drug loading efficiency (DL).

The three-dimensional effect surface curves of A, B, and C on Y1, Y2 are shown in **Figure 6**. Using Design-Expert 8.0.6, we predicted that the best formulation of CHELP was as follows: CHOL : HSPC, 0.87:1 (w/w); drug–lipid ratio, 0.21; and drug incubation time, 26.54 min. The drug loading and particle size of CHELP were predicted to be 16.88% and 123.3 nm, respectively. Thus, three parallel experiments were conducted, and the deviation between the measured and predicted values was small, showing that the optimal prescription parameters were reliable and feasible (**Table 7**).

**Table 7:** Comparison of predicted and experimental value (n = 3).

Sample	Experimental value of size (nm)	Predicted value of size (nm)	Prediction error of size (%)	Experimental value of DL (%)	Predicted value of DL (%)	Prediction error of DL (%)
1	128.0	123.3	3.81	16.81	16.88	−0.39
2	126.2	123.3	2.35	16.89	16.88	−0.07
3	124.2	123.3	0.73	16.29	16.88	3.47
Mean absolute difference	126.13	123.3	2.30	16.67	16.88	1.31

### Characterization of CHELP

**Figure 7:**
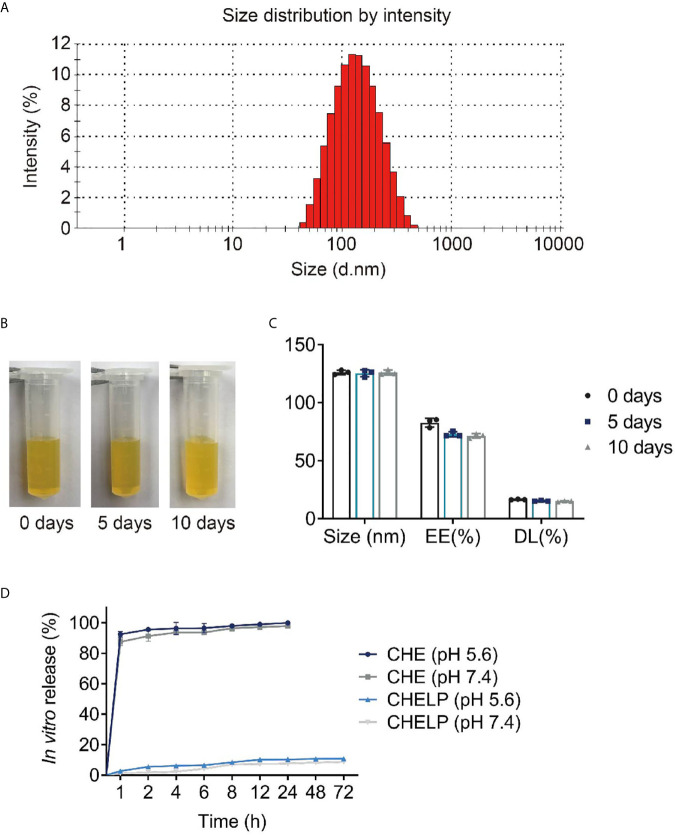
Characterization of CHELP. **(A)** The size distribution of CHELP; **(B, C)** The stability of CHELP; **(D)** *In vitro* release of CHELP.

The average size of CHELPs was 126.1 ± 1.9 nm; PDI was 0.233 ± 0.02, which was less than 0.3 (**Figure 7A**); and the zeta potential of CHELPs was −40.1 ± 2.20 mV. The entrapping efficiency and drug loading efficiency were 83.94 ± 1.72% and 16.67 ± 0.32%, respectively. After storage at 4°C for 10 days, no obvious difference in particle size and obvious massive drug leakage were observed (**Figures 7B, C**). The entrapping efficiency and drug loading efficiency were 71.48 ± 1.95% and 15.01 ± 0.41%, respectively, showing the good stability of CHELP. According to the result of the *in vitro* release study (**Figure 7D**), the 72 h releasing rate of CHELP was less than 20%, and CHE was released up to 100% in 8 h, which showed sustained release effect of CHELP *in vitro* compared with free drug.

### *In Vivo* Pharmacokinetic Study of CHELP

**Figure 8:**
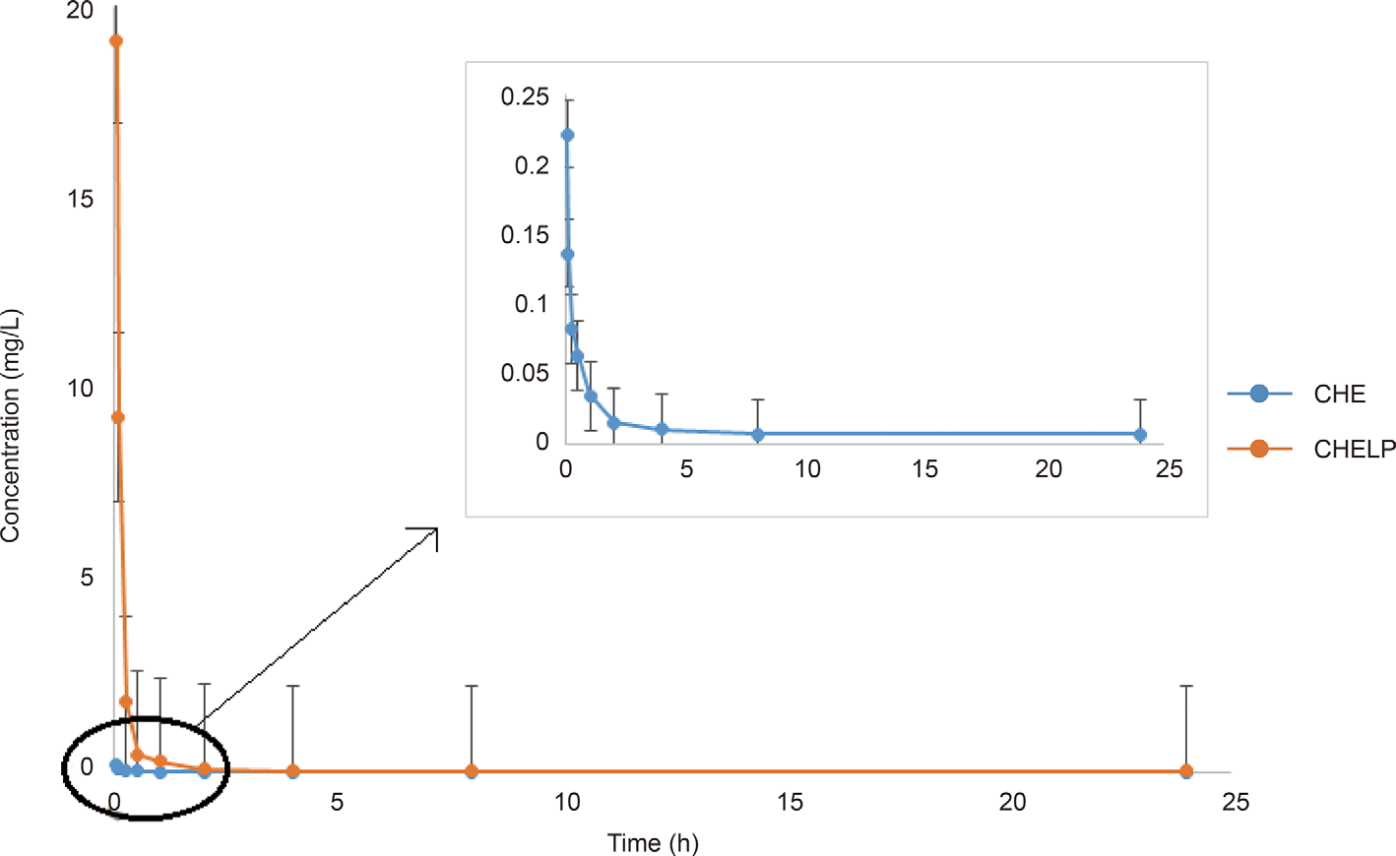
Blood concentration–time curve of CHE and CHELP (n = 3).

The results of the blood concentration–time curve of CHE and CHELP are shown in **Figure 8**. DAS 2.0 was used to process and analyze blood concentration data, and corresponding pharmacokinetic parameters were obtained. The parameters are shown in **Table 8**. The results showed that when CHE free drug and CHELP were injected into the tail vein at the same dose (6 mg/kg), the AUC_0-t_ of CHELP was 4.349 mg/L * h, which was nearly 15 times that of the free drug. The highest plasma concentration (C_max_) of CHELP, 19.122 mg/L, was significantly higher than that of the free drug. Meanwhile, the CHELP clearance rate of CL was 1.353 L/h/kg, which was significantly lower than that of the free drug. As can be seen from the pharmacokinetic parameters, CHELP has a lower *in vivo* clearance rate and higher absorption concentration than the free drug, thereby increasing the bioavailability of CHE.

**Table 8:** Pharmacokinetic parameters of CHE and CHELP.

Parameters	CHE	CHELP
AUC_0–t_ (mg/L · h)	0.282	4.349
AUC_0–∞_ (mg/L · h)	0.291	4.435
C_max_ (mg/L)	0.221	19.122
CL (L/h/kg)	20.624	1.353
V (L/kg)	173.455	12.832
T_1/2_ (h)	5.828	6.573

### *In Vivo* Antitumor Effect

**Figure 9:**
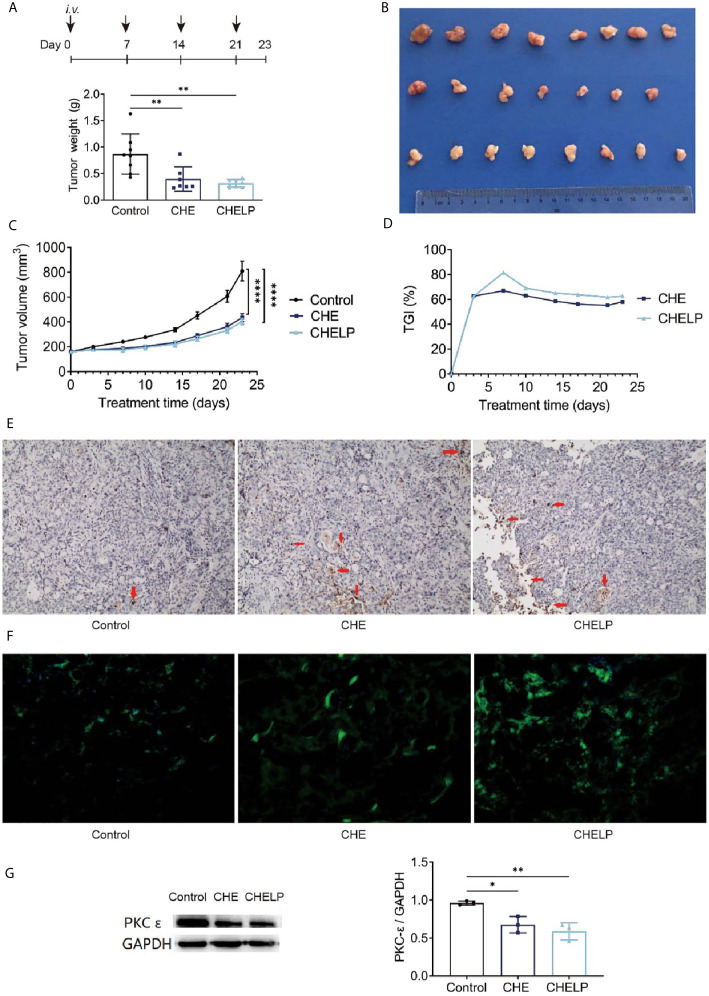
Antitumor effect of CHE and CHELP on HCC827 tumor-bearing nude mice. (Saline group n = 8; CHE treatment group n = 7; CHELP treatment group n = 8). **(A)** Tumor weight; **(B)** the record of tumors; **(C)** tumor growth; **(D)** tumor growth inhibition; **(E)** apoptosis of tumors; **(F)** ROS generation detection in tumors; **(G)** the expression of PKC-*ϵ* in tumors (n = 3). (****p < 0.0001, **p < 0.01, *p < 0.05).

CHE and CHELP had a good therapeutic effect on HCC827 tumor-bearing nude mice and inhibited tumor growth (**Figures 9A–C**). The CHELP inhibition of tumor was slightly better than the free medicine tumor suppression effect. The average tumor weight of the CHELP treatment group showed small differences, whereas the tumor weight of the CHE group was not uniform. The tumor inhibition rate of the CHELP group was slightly higher than that of the CHE treatment group, reaching approximately 80% (**Figure 9D**).

### Induced Apoptosis Through the Regulation of ROS Generation and PKC-ϵ Expression

The number of apoptotic tumor cells was observed in the CHE and CHELP groups, and the degrees of apoptosis were very close (**Figure 9E**). CHE and CHELP induced the apoptosis of tumor cells *in vivo* and showed similar effects. Both effectively increased ROS generation in the tumor tissues and induced the apoptosis of tumor cells (**Figure 9F**). In this assay, CHELP showed a better pro-apoptotic effect than CHE. Compared with the control group, the CHE and CHELP groups showed significantly decreased expression levels of protein PKC-*ϵ* in the tumor tissues of CHE and CHELP groups, indicating that CHE and CHELP can inhibit the expression of protein PKC-*ϵ in vivo*, and their inhibitory effects were close (**Figure 9G**). CHE and CHELP can inhibit tumor growth by inhibiting the expression of protein PKC-*ϵ* in nude mice.

### Safety Evaluation and Histopathological Analysis

**Figure 10:**
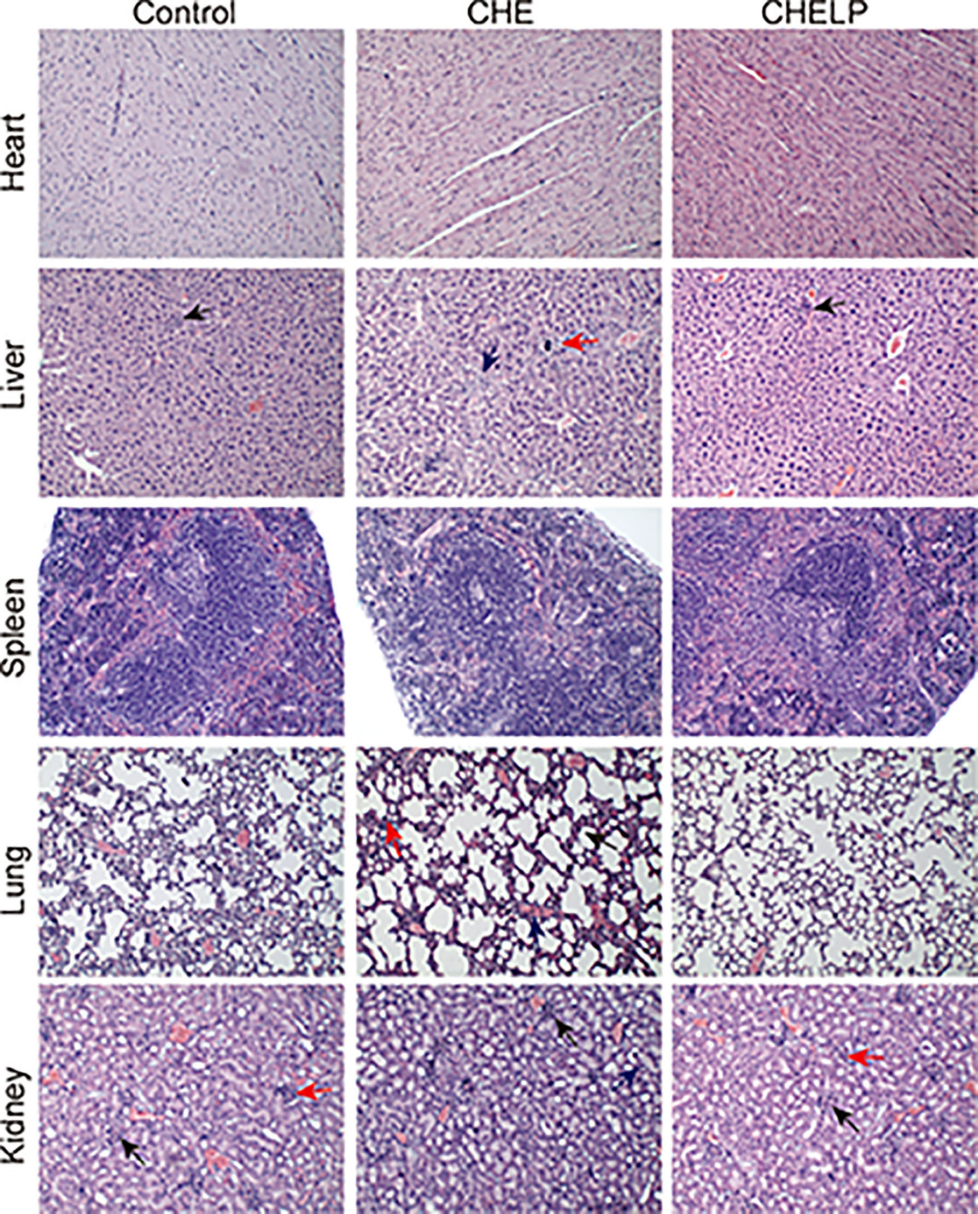
Pathological evaluation. (1) Liver, a small amount of inflammatory cells infiltrate (↑); (2) Lung, alveolar septa slightly thickened (↑), inflammatory cell proliferation (↑), slight congestion in the blood vessels (↑); (3) Kidney, the renal microcysts become smaller or disappear (↑), glomerular atrophy (↑), the renal tubules are marked by clear tubules (↑).

**Figure 11:**
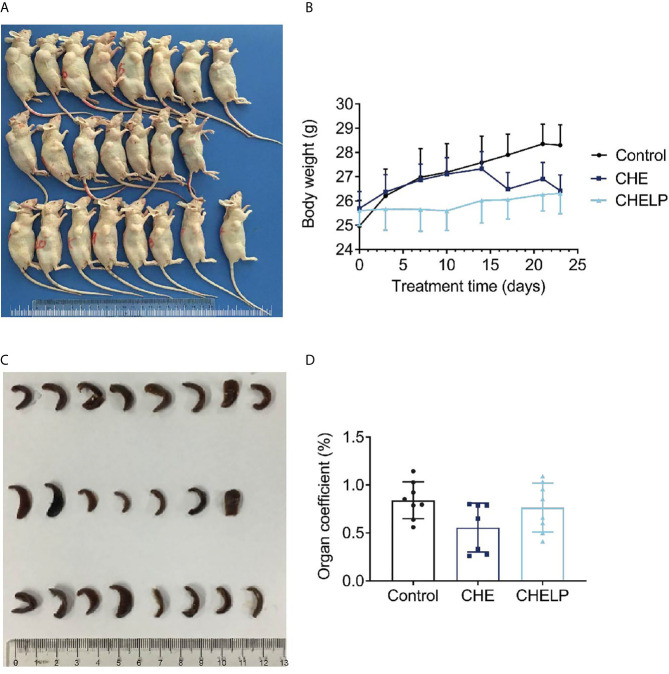
Safety evaluation. **(A)** The picture of nude mice in different groups after sacrificed. **(B)** Body weight; **(C)** spleen morphology; **(D)** organ coefficient of spleen. (Saline group n = 8; CHE treatment group n = 7; CHELP treatment group n = 8).

Three mice in the CHE treatment group died during the treatment, indicating that the CHE free drug exerted a certain level of toxicity to nude mice and caused these deaths (**Figures 10** and **11A**). The backs of the nude mice in the CHE group were thin and prominent, and the tails of the nude mice were stiff and black. Some of them even had broken tails, which were generally pathological. In the CHELP group, the overall morphology of the nude mice was healthy, and the tail was smooth without breakage, indicating that CHELP was safer for nude mice than CHE (**Figure 11A**). According to the body weight data of the nude mice, the body weight of the nude mice in the CHELP treatment group was stable and increased slightly in the later period (**Figure 11B**). In the CHE group, significant weight loss was observed in the later period. The spleens of the mice in the CHE treatment group shrank and had obvious lesions (**Figure 11C**). The organ coefficient of the spleen decreased in the CHE treatment group, whereas that of the CHELP treatment group remained close to that of the control group (**Figure 11D**).

## Discussion

CHE inhibits proliferation and induces apoptosis activities on various human tumor cell lines (20, 30, 31). However, it has a certain level of toxicity (22, 32), which probably causes challenges and problems for its applications. In this paper, we studied the *in vitro* and *in vivo* effects of CHE on tumor inhibition and discussed its possible action mechanism. CHE liposome delivery system was prepared for the purpose of toxicity reduction.

*In vitro* antitumor effect of CHE on non-small cells lung cancer cell line HCC827 was confirmed, and glycolysis and ROS/PKC-*ϵ*/caspase-3 pathway might be the underlying mechanism. MTT and CCK-8 assay showed that CHE suppressed the viability and proliferation of HCC827 cells in a dose-dependent manner, and the wound-healing and trans-well migration assays showed the restricted migration and invasion capacity of HCC827 after treatment with CHE. CHE arrested cell cycle in the G2/M phase of renal cell carcinoma (24) and induced apoptosis through the generation of ROS (21) and ROS-caused endoplasmic reticulum stress (24), indicating that ROS is the critical factor in cell apoptosis. The increase in intracellular ROS resulted in cytotoxicity to cells by acting on proteins and damaging DNA and mediated rapid apoptosis (27, 33). Caspase 3 is another important indicator of early apoptosis and can reflect the pro-apoptotic effects of drugs on cells and participate in the regulation of apoptosis through various pathways (28, 29). The relation between ROS and PKC has been reported in many studies (34, 35). We found that CHE impeded cell growth by blocking cells in the G2/M phase. The ECAR results showed that the glycolysis of HCC827 was inhibited by CHE in a dose-dependent manner, reducing cell energy supply. Moreover, CHE elevated ROS generation and down-regulated the PKC-*ϵ* and caspase 3, thereby inducing cell apoptosis and inhibiting NSCLC HCC827 cell growth. However, when the PKC activator PMA was added, the inhibition of CHE on the protein PKC and the promotion of caspase 3 activity were blocked. Thus, the ROS/PKC-*ϵ*/caspase 3 pathway is a possible mechanism that CHE uses to inhibit the growth of HCC827 tumor cells, and this finding might provide insight into the mechanism of CHE’s antitumor effect.

For the reduction of CHE toxicity, the liposome of CHE was designed and optimized. We referred to the prescription of Doxil, the first PEGylated nano-drug approved by the FDA, which shows prolonged circulation time and clinical safety, and reduces the cardiotoxicity of doxorubicin (36), which is close to our research purpose. In our study, the CHELP prescription combined with the same lipid materials as Doxil was used and optimized through the central composite design/response surface method. CHELP was optimized to receive uniform particle size distribution, good encapsulation rate, drug loading efficiency, good stability, and sustained release effect. Doxil can be released sustainably at less than 20% in 24 h (37). CHELP showed about one-fifteenth of the *in vivo* clearance rate and 86 times the absorption concentration of free drug CHE, exerting a good therapeutic effect on HCC827 tumor-bearing nude mice.

CHELP, PEGylated liposomes, selectively accumulated drugs at the tumor site through enhanced permeability, and retention effects after a passive targeting strategy was used (38). CHELP induced tumor cell apoptosis and inhibited tumor growth by inhibiting the expression of the protein PKC-*ϵ* and elevating ROS generation possibly through the same action mechanism as that in the *in vitro* study. As expected, CHELP significantly reduced the toxicity of CHE after being encapsulated by lipid materials. In the CHE group, three mice died during the treatment, and the morphology of other mice was pathological, with a low organ coefficient. The differences between the complicated inner environment of organism and single cell lines might be the reason that CHE did not exhibit obvious cytotoxicity to HL-7702 and HEK-293 but eventually led to the death of mice. By contrast, CHELP was safer for the nude mice and reduced the toxicity of CHE and protected the lung and spleen from pathological changes in organs.

There are still some problems to be improved in this article. First of all, the initial purpose of CHELP is to achieve long circulation and reduce the toxicity of CHE. Although the preparation successfully reduced the toxicity of CHE *in vivo* and avoided the death of experimental animals, pharmacokinetic studies showed that the half-life of the drug was not significantly improved. Theoretically, PEG in CHELP lipid material DSPE-PEG2000 enhanced the hydrophilicity of liposomes, prevented the recognition and adsorption of proteins in the blood, and extended circulation time (23). CHELP showed a good sustained-release effect *in vitro*. However, the *in vivo* results were not as expected, which may be caused by the complexity of the internal environment, and need to be investigated subsequently. Another possible explanation is that the concentration of free agents detected *in vivo* is too low. The pharmacokinetic study of CHE and its liposomes (39) showed that the half time (5.66 h) of CHE liposomes was similar to ours after oral administration to rats, but the half-life of free drugs was much lower than the data in our study. Therefore, the sustained release and long circulation effects of liposomes *in vivo* may be masked. Secondly, although the formulation process has been optimized, the loading of CHE still needs to be further improved. Besides drug-to-lipid ratio, the proportion of cholesterol and hydrogenated soybean phosphatidylcholine, and drug incubation time, other factors including liposome internal volume, ammonium-sulfate gradient, and lipid bilayer physical state, also affect the active-loading process.

## Conclusion

CHE probably inhibited NSCLC cell growth through the ROS/PKC-*ϵ*/caspase 3 pathway and regulated the level of cell glycolysis and metabolism. CHELP exerted a sustained release effect and had a higher drug concentration in blood than the free drug. Additionally, CHELP significantly inhibited tumor growth in HCC827 tumor-bearing nude mice, reduced the toxicity of CHE, and improved the drug safety. For further study, we are looking forward to preparing active-targeting CHE liposomes for enhanced antitumor effect and comprehensive study.

## Data Availability Statement

The original contributions presented in the study are included in the article/supplementary material. Further inquiries can be directed to the corresponding authors.

## Ethics Statement

All animal experiments were approved by the Laboratory Animal Welfare and Ethics Committee of Shanghai University of Traditional Chinese Medicine (approval no. PZSHUTCM18122114 for nude mice and no. PZSHUTCM19092004 for ICR mice) and were performed strictly in accordance with the Guide for the Care and Use of Medical Laboratory Animals. Written informed consent was obtained from the owners for the participation of their animals in this study.

## Author Contributions

JW and YS contributed significantly to complete manuscript preparation. NZ and NL contributed to the constructive discussions. BW and CL contributed to the conception of the review. All authors contributed to the article and approved the submitted version.

## Funding

This work was supported by grants from Program of Shanghai Committee of Science and Technology, China (Grant No. 17401902300).

## Conflict of Interest

The authors declare that the research was conducted in the absence of any commercial or financial relationships that could be construed as a potential conflict of interest.
